# Prevalence of *Helicobacter pylori* Infection and Diagnostic Methods in the Middle East and North Africa Region

**DOI:** 10.3390/medicina56040169

**Published:** 2020-04-09

**Authors:** Faten A. S. Alsulaimany, Zuhier A. Awan, Ahmad M. Almohamady, Mohammed I. Koumu, Bassam E. Yaghmoor, Sameh S. Elhady, Mahmoud A. Elfaky

**Affiliations:** 1 Department of Biological Sciences, Faculty of Science, King Abdulaziz University, Jeddah 21589, Saudi Arabia; faalsulaimany@kau.edu.sa; 2 Department of Clinical Biochemistry, Faculty of Medicine, King Abdulaziz University, Jeddah 21589, Saudi Arabia; zawan@kau.edu.sa (Z.A.A.); ha.mody1416@hotmail.com (A.M.A.); moh.komo@hotmail.com (M.I.K.); bas_yag@hotmail.com (B.E.Y.); 3 Department of Natural Products, Faculty of Pharmacy, King Abdulaziz University, Jeddah 21589, Saudi Arabia; ssahmed@kau.edu.sa; 4 Department of Pharmacognosy, Faculty of Pharmacy, Port Said University, Port Said 42526, Egypt

**Keywords:** *Helicobacter pylori*, prevalence, genotypes, diagnosis, MENA

## Abstract

*Background and Objectives:Helicobacter pylori* (*H. pylori*) infection is common worldwide and may cause gastroduodenal complications, including cancer. In this review, we examine the prevalence and distribution of various *H. pylori* genotypes and the risk factors for *H. pylori* infection, particularly in the Middle East and North Africa (MENA) region. We also introduce different global screening methods and guidelines and compare them to those currently in use in the MENA region. *Materials and Methods:* We searched the Google Scholar, PubMed, and Saudi Digital Library (SDL) databases for clinical trials and articles published in English. The data collection was mainly focused on MENA countries. However, for *H. pylori* genotypes and diagnostic methods, studies conducted in other regions or reporting global practices and guidelines were also included to allow a comparison with those in the MENA region. We also included studies examining the prevalence of *H. pylori* infection in healthy participants. *Results:* *H. pylori* infection is highly prevalent in the MENA region, mainly because of the accumulation of risk factors in developing countries. Herein, we highlight a lack of good quality studies on the prevalence of various *H. pylori* genotypes in the MENA region as well as a need for standard diagnostic methods and screening guidelines. Due to the complications associated with *H. pylori*, we recommend routine screening for *H. pylori* infection in all gastroenterology patients admitted in the MENA region. *Conclusion:* Concerted effort will first be required to validate affordable, non-invasive, and accurate diagnostic methods and to establish local guidelines with adapted cut-off values for the interpretation of the test results.

## 1. Introduction

*Helicobacter pylori* (*H. pylori*) (also was known as *Campylobacter pylori* or *Campylobacter pyloridis*) is a gram-negative, microaerophilic bacterium with a helical, curved shape (often referred to as an S-shape). *H. pylori* has multiple polar-sheathed flagella, which are involved in its motility and invasion mechanisms [1,2,3]. In animals, Bizzozero [4,5] was probably first in the second part of the 19th century to report the presence of such organisms in the gastrointestinal tract. He was the first person who observed and described spiral organisms in the stomach of dogs.

A century ago, Jaworski [6,7] at Cracow University detected a spiral bacteria named *Vibrio rugula*, in the sediment after gastric washing from patients with gastric cancer (GC) and over a quarter of century since Marshall and Warren drew attention to the spiral bacteria, *Helicobacter pylori*, as a pathogen in various gastric diseases. In the 1984, *H*. *pylori* was isolated and cultured by Robin Warren and Barry Marshall [8]. *H. pylori* is found primarily in the human gastric mucosa, its natural habitat, where it remains close to epithelial cells. Indeed, *H. pylori* is attracted to the gastric mucus layer, which offers cover and protection from the high acidity in the stomach and promotes better cell motility [9].

Numerous studies have shown that *H. pylori* infection is the leading bacterial cause of both malignant and non-malignant gastroduodenal diseases and is also involved in extra-gastroduodenal disorders. Among these, the most common disorders are peptic and duodenal ulceration, acute and chronic gastritis (which may lead to atrophic gastritis), and gastric adenocarcinoma (B-cell gastric lymphoma and mucosa-associated lymphoid tissue (MALT) lymphoma) [10,11]. However, most of the risk reduction due to improved socio-economic status (even in the absence of specific preventive strategies) is thought to stem from reduced *H. pylori* infection rates [12]. Due to the variety of risk factors present in developing countries, infection with multiple *H. pylori* genotypes is highly prevalent in the Middle East and North Africa (MENA) region. There are 19 countries that are generally considered part of the MENA region according to the World Bank and the United Nations. These are Algeria, Bahrain, Egypt, Iran, Iraq, Israel, Jordan, Kuwait, Lebanon, Libya, Morocco, Oman, Palestine, Qatar, Saudi Arabia, Syria, Tunisia, United Arab Emirates, and Yemen.

The pattern of infection is an early childhood acquisition of *H. pylori* (30–50%) that reaches over 90% during adulthood in developing countries in MENA region due to the poor socioeconomic status and overcrowded conditions [13]. The data presented on the prevalence of *H. pylori* in the Middle East are not completely satisfactory, it does again suggest the critical role of socio-economic development in determining *H. pylori* prevalence, particularly in childhood. Few studies and reviews on prevalence and diagnostic methods of *H. pylori* in MENA region are published, therefore it is important to summarize present knowledge on *H. pylori* in the region. In this review, we report the prevalence and distribution of different *H. pylori* genotypes in the MENA region, as well as the risk factors for *H. pylori* infection. We also present different screening methods available for the diagnosis *H. pylori* infection and global guidelines, while comparing them to those currently in use in the MENA region.

## 2. Methods

In this review, we searched the Google Scholar, PubMed, and Saudi Digital Library (SDL) databases for about 90 studies among clinical trials, review and full research articles published in English from 1984 until 2020. The studies included in this review were primarily conducted in the countries of the MENA region. However, for *H. pylori* genotypes and diagnostic methods, studies conducted in other regions were also included to allow the comparison between the global practices and guidelines and those in use in the countries of the MENA region. The keywords used to interrogate the databases included “*Helicobacter pylori*” in combination with “prevalence,” “genotype”, “risk”, “screening”, “diagnosis”, “guidelines”, “symptoms” and “complications” as well as “world”, “global”, “Europe”, “Arab”, “Saudi”, “Middle East”, “North Africa” and other specific region or country names. All articles were assessed for pertinence before inclusion. Studies examining the prevalence of *H. pylori* infection in healthy participants were also included.

## 3. Results and Discussion

### 3.1. Prevalence of H. pylori Infection

***Worldwide****—H. pylori* infection is common worldwide and can be either symptomatic or asymptomatic. A recent review, including global studies published between 2000 and 2014, examined the prevalence of *H. pylori* infection in various African, South American, Asian, and European general adult populations. This review showed that the prevalence of infection varied by country (18.2–60.5% in Uganda, 68.6% among pregnant women in Chile, 80% in Bolivia, 83.4% in China, 29.4–54.5% in Japan, 76.5% and 47.9% among the Yami and Han ethnic groups in Taiwan, respectively, 72.1% in Italy, and 84.2% in Poland). The authors of the review also identified several risk factors associated with *H. pylori* infection, including low socioeconomic status, vegetarian diet (in the Chinese study), environmental factors (e.g., contaminated or untreated water), poor health, crowded living conditions, and day care center attendance (leading to person-to-person transmission among children) [14]. Another systematic review published in 2017 compiled 184 global studies conducted between 1970 and 2016 in a total of 62 countries [15]. The selected samples were representative of the general population, and the prevalence of *H. pylori* infection among all participants was 48.5%. While the infection was widely spread in developing countries (70.1% in Africa, 69.4% in South America, and 66.6% in Western Asia), the prevalence was significantly lower in developed countries (24.4% in Oceania, 34.3% in Western Europe, and 37.1% in North America) [15].

***MENA region***—The prevalence of *H. pylori* infection among the countries of the MENA region varies widely ranging from 7–50% in young children and going up to 36.8–94% in adults [16]. Data about prevalence of *H. pylori* infection among the MENA region are summarized (Table 1).

### 3.2. Risk Factors for H. pylori Infection

The risk factors for *H. pylori* infection have been investigated in several countries of the MENA region. Age (>10 years), low socioeconomic status, low level of education, married participants, number of residents per household, bed sharing, drinking municipal or tank water, eating outside the home, living in rural areas, smoking, alcohol consumption, type 2 Diabetes Mellitus, chronically dyspeptic patients, eating raw vegetables or spicy food and having two affected parents were identified as risk factors in the majority of studies [17,28,31,34,35,38,39,40].

However, in a study conducted in Mecca, Khan et al. observed a negative correlation between the presence of antibodies for *H. pylori* and smoking in females (antibodies were detected in 8% and 91% of the smokers and non-smokers, respectively) [32]. Examining other risk factors, they did not find a significant association between infection and eating spicy food or drinking-water source. In the same study, they also examined a potential correlation between eating vegetables and infection but found no evidence. Indeed, this study should have considered the difference between raw and cooked vegetables, together with other dietary habits of the participants.

In another study, Bassily et al. found that a higher level of education was positively correlated with the risk of infection in mothers, even after adjusting for the age of the mother [19].

In a study conducted in Iran, the rate of infection increased with age and number of family members but was negatively correlated with the level of education and marital status [22].

Also in Mansour-Ghanaei et al. study, they established that there was no association between *H. pylori* infection and these and other factors, including living areas, level of education of the parents, and monthly income. [21].

While the majority of the studies included in this review did not find a significant relationship between gender and rate of infection, two studies by Soltani et al. [22] and Al-Balushi et al. [29] found that seropositivity was more widespread in male than in female participants.

Beyond general improvements in socio-economic status leading to improved health and lower *H. pylori* infection rates, specific local strategies are needed to further reduce the number of incident cases and deaths due to stomach cancer, and these should be tailored to each country’s risk factor profile. Targeting the risk factors that affect stomach cancer incidence and mortality (such as smoking and diet), in addition to country specific feasible and cost-effective interventions aimed at lowering *H. pylori* infection rates, early detection of suspected cases, and improved access to standard treatment facilities, can be among such strategies. By providing annual updates to regional and country-level stomach cancer estimates, future iterations of global burden of disease study will be useful for monitoring the success of such strategies [12].

### 3.3. Prevalence of H. pylori Genotypes and Their Correlation with Disease

***Worldwide***—In an extensive review aimed at studying the prevalence of *H. pylory vacA* alleles in 24 countries worldwide, Van Doorn et al. found that 89% of the strains were subtype s1a in Northern and Eastern Europe, whereas 89% were subtype s1b in Southwest Europe (Spain and Portugal) [41]. Moreover, subtypes s1a and s1b were equally prevalent in France and Italy, and nearly equally present in North America. Most of the s1 strains isolated in Middle and South America were subtype s1b, while 77% of the s1 isolates in East Asia were subtype s1c. While subtype m1 was most common in the Iberian Peninsula (86.2%), subtypes m1 and m2a were equally present across North and South America. Subtype m2b was only detected among s1c strains found in East Asia. Globally, *cagA* +/*vacA*s1 genotypes were associated with peptic ulcer disease. The presence of *cagA* was significantly associated with *vacA*s1, while *vacA*s1 was associated with *vacA*m2. Specific *vacA* subtype m alleles were also preferentially associated with certain diseases, with subtypes m1 and m2 more prevalent in carcinomas and peptic ulcers, respectively.

***MENA region***—*H. pylori* main virulence genes are *cagA*, *vacA* (which has multiple subtypes), and *iceA*. Numerous regional and global studies have highlighted consistent associations between particular genotypes and diseases: *cagA* and *vacA* (particularly subtypes s1 and m2) with gastritis; *cagA*, *vacA*s1m1, and *iceA* with ulcers; and *cagA* with cancers. However, other genotypes were only considered in a small number of studies. For example, *ureA* in Saudi Arabia, *babA2* in Iran, *dupA* in Iraq and Iran, and *oipA* in Tunisia and Iran have all been associated with ulcers (although some studies reported inconsistent results). In addition, *cagE* in Iraq, Iran, and Israel, and *vacA*i1 in Egypt have been associated with cancer, while *cagE*, in combination with other genes, was also associated with non-ulcer dyspepsia in Iran [42].

In Kuwait, the study by Al-Qabandi et al. is the only included one to examine the correlation between the prevalence of *H. pylori* genotypes and the nationality of the patients [43]. While the overall prevalence of *cagA*+ strains was 53%, a significant variation was observed with a prevalence of ~41%, 25%, 75%, and 87% in patients from the Gulf region, Egypt, India, and Bangladesh, respectively. Although the statistical significance was not considered, a substantial variation was also reported for *vacA* subtypes. While s1 subtypes were more prevalent in patients from India and Bangladesh, s2 subtypes were more prevalent in African patients (e.g., patients from Egypt and Somalia).

In Israel, a study by Muhsen et al. examined the prevalence and correlation of *H. pylori* infection according to *cagA* phenotype among the ethnically diverse population groups of Jerusalem. A cross-sectional study was undertaken in Arab (N = 959) and Jewish (N = 692) adults, randomly selected from Israel’s national population registry in age-sex and population strata. Sera were tested for *H. pylori* immunoglobulin G (IgG) antibodies. Positive samples were tested for virulence IgG antibodies to recombinant *cagA* protein, by enzyme-linked immunosorbent assay. Multinomial regression models were fitted to examine associations of sociodemographic factors with *H. pylori* phenotypes. *H. pylori* IgG antibody sero-prevalence was 83.3% (95% confidence interval (CI) 80.0–85.5%) and 61.4% (95% CI 57.7–65.0%) among Arabs and Jews, respectively. Among *H. pylori* positives, the respective *cagA* IgG antibody sero-positivity was 42.3% (95% CI 38.9–45.8%) and 32.5% (95% CI 28.2–37.1%) [44].

### 3.4. Diagnostic Methods for H. pylori

The different methods available to detect *H. pylori* infection can be classified according to their invasiveness (invasive or noninvasive) and timing relative to treatment (before or after treatment). The choice of one method over another is based on multiple factors, including the prevalence of infection in the population, the age of the patient, and the cost of the procedure.

#### 3.4.1. Invasive Methods

***Histology*:** This method has the advantage of also detecting changes in the mucosa. Several studies recommend taking two biopsies each from the corpus and antrum of the stomach [45,46,47,48], but the current gold standard is the updated Sydney classification system from Dixon et al. [49], which advises collecting specimens from five or more sites including the incisura angularis. However, to date, the available studies do not demonstrate any significant advantage of the latter approach [50,51]. Moreover, the sensitivity and specificity of histology vary between 53% and 90%, depending on the physician’s experience and density of colonization [52]. It is also important to note that this method requires endoscopic intervention, which limits its use.

***Fluorescent in-situ hybridization (FISH)*:** This method can also be used to detect specific factors or features of the bacterium, such as drug resistance [53,54]. The probes most commonly used target the 16 S and 23 S rRNA genes. FISH can be used to locate *H. pylori* within the gastric mucosa [55]. Importantly, this method is rapid, taking only three hours to detect *H. pylori* and clarithromycin resistance, but is expensive and cannot be used in clinical practice.

***Culture*:** Although this method can be less sensitive, it requires minimally-invasive procedures, such as gastric juice sampling or the string test [56,57]. When performed with the proper media and reagents, culture usually has both high sensitivity (>90%) and specificity (100%) [58]. However, both sensitivity and specificity can be affected by certain conditions (e.g., bleeding reduces the sensitivity to 40%) [59] or age (e.g., in a group of pediatric patients the sensitivity and specificity were 95.8% and 96.4%, respectively) [60]. Culture is usually used in clinical practice after two treatment failures. However, in light of the increasing rate of *H. pylori* treatment failure, it would be advisable to perform culture earlier (i.e., before two treatment failures) [52]. It is important to note that culture is also susceptible to variation depending on the pathologist’s experience, the specimen quality, and the transport media [61].

***Polymerase chain reaction (PCR)*:** PCR can be performed on specimens collected from both invasive and noninvasive procedures. It is often used for small samples, which contain a limited number of bacteria. PCR can also be used to detect clarithromycin resistance [62] and is even useful after a prolonged period, when culture is not applicable. However, PCR can detect DNA from both live and dead bacteria, which may produce false positive results.

***Rapid urease test(RUT)*:** RUT it based on the breakdown of urease and the resulting change in pH. The sensitivity and specificity of this method are both affected by the presence of blood, while an increased storage time of the specimen reduces the specificity. There is also a risk of false negative results when the patient takes antibiotics or suffers from a reduction of gastric acid or achlorhydria [52]. There is good availability of commercial RUTs (e.g., CLOtest, HpFast, PyloriTek), which have both high sensitivity (85–95%) and specificity (95–100%) [63]. This diagnostic method is also relatively fast, with results obtained between a few minutes and 24 h, depending on the density of bacteria.

#### 3.4.2. Non-Invasive Methods

***Serology*:** Among the different types of serologic test available, the most commonly used is the enzyme immunoassay (EIA). EIA has a sensitivity and specificity of 60–100%, and the quality of the test must be evaluated for each population to fix cut-off values [52]. This method should be particularly considered for patients who previously used proton pump inhibitors (PPIs), or suffered from bleeding ulcers or gastric atrophy. One drawback of serology is the persistence of antibodies even after eradication of the infection. However, the antibodies against the heat shock protein 60 (HSP60) have been shown to decline after a relatively short treatment time (one month), while a significant correlation has been observed between the level of anti-HSP60 antibodies and the histological data [64]. Importantly, serology is an affordable method and can be useful to exclude infection [65]. Serologic tests using urine or saliva samples have also been considered, but their usefulness was limited by the reduced accuracy of the results [66].

***Urea breath test (UBT)*:** This method relies on the urease activity of *H. pylori*, which converts ingested ^13^C- or ^14^C-labeled urea into CO_2_ and NH_3_. UBT is usually used as a follow-up method of detection after 4–6 weeks of treatment. UBT is also affordable and has both high sensitive and specific (>95% in most well-designed studies) [67]. However, UBT requires access to a nuclear medicine department and the device used to run the tests can be expensive. This method is also subject to false negative results, which can happen in patients treated with antibiotics or PPIs, and patients suffering from corpus-predominant gastritis [68]. In general, UBT is considered more sensitive than biopsies, particularly in cases of moderate or patchy distribution of bacteria. ^14^C-urea is radioactive, and its use is contraindicated in children, pregnant women, and probably women of childbearing age [69].

***Stool antigen test (SAT)*:** SAT is a type of EIA, and is used both for diagnosis and to assess the response to treatment of a *H. pylori* infection. The different SATs evaluated have shown both varying sensitivities (48.9–100%) and specificities (87–94.4%) [52]. While SATs using monoclonal antibodies are superior to SATs using polyclonal antibodies in both pre- and post-treatment conditions [70,71], SATs are comparable to UBTs in pre-treatment but inferior in post-treatment [52].

#### 3.4.3. Guidelines for the Diagnosis of *H*. *pylori* Infection

***Worldwide***—According to the global guidelines, more than one positive test is required for the diagnosis of *H. pylori* infection, except in cases of duodenal ulcer. The World Gastroenterology Organisation (WGO) global guidelines by Hunt et al. indicated that serology is less accurate than SAT in areas with low prevalence of *H. pylori* infection, where a negative test result is more valuable. Indeed, false positive results were considered dangerous because they can lead to the unnecessary administration of antibiotics. Conversely, serology was considered sufficient for diagnosis in areas with high prevalence of *H. pylori* infection, such as the MENA region. In areas with high rates of ulcer and gastric cancer, the WGO global guidelines also recommended to adopt an empirical test-and-treat approach or to perform an initial endoscopy for gastroenterology patients, rather than initiating treatment with PPIs regardless of the underlying causes. Importantly, serologic tests detecting antibodies against the FliD protein, an essential component of bacterial flagella, demonstrated very high specificity and sensitivity (>95% against various standards) compared to other antigens. In addition to its quality, this test was affordable and was therefore considered to be an ideal tool for screening *H. pylori* infection in developing countries with a high prevalence [72].

In Japan, Asaka et al. added SAT to the list of noninvasive methods given in the guidelines for *H. pylori* diagnosis [73]. Moreover, the Second Asian Pacific Consensus guidelines for *H. pylori* infection indicated that SAT was an acceptable diagnostic tool, while UBT was the most accurate of the noninvasive methods and serology had a limited role in the management of *H. pylori* infection, due to its highly variable accuracy [74].

In Germany, the guidelines published by Fishbach et al. recognized all the diagnostic methods for *H. pylori* mentioned in this review [75].

Regarding the indications for *H. pylori* diagnosis, Fishbach et al. considered that gastric and duodenal ulcers or MALT lymphoma were absolute indications, while functional dyspepsia, cancer prevention, initiation of long-term treatment with nonsteroidal anti-inflammatory drugs (NSAIDs), or underlying gastroduodenal complications accompanied by the use of NSAIDs or acetylsalicylic acid were relative indications [75]. In addition, the international consensus recommendations for the management of patients with nonvariceal upper gastrointestinal bleeding included histology or UBT for the detection of *H. pylori*, 4–8 weeks from the bleeding episode and only if the initial index endoscopy was negative [76].

In Western Australia, Wise et al. reported that, UBT is one of the most accurate and reliable non-invasive methods for diagnosing active *H. pylori* infection [77].

In a study done on behalf of the EHMSG (European Helicobacter and Microbiota Study Group) and Consensus panel by Malfertheiner et al. concluded that, UBT is the most investigated and best recommended non-invasive test in the context of a “test-and-treat strategy”. Monoclonal SAT can also be used. Serological tests can be used only after validation. Rapid (“office”) serology tests using whole blood should be avoided in this regard. In clinical practice when there is an indication for endoscopy, and there is no contraindication for biopsy, the rapid urease test (RUT) is recommended as a first-line diagnostic test. In the case of a positive test, it allows immediate treatment. One biopsy should be taken from the corpus and one from the antrum. RUT is not recommended as a test for *H. pylori* eradication assessment after treatment. For assessment of *H. pylori* gastritis, a minimum standard biopsy setting is two biopsies from the antrum (greater and lesser curvature 3 cm proximal to the pyloric region) and two biopsies from the middle of the body. Additional biopsy from the incisura is considered for detection of precancerous lesions. Most cases of *H. pylori* infection can be diagnosed from gastric biopsies using histochemical staining alone. In cases of chronic (active) gastritis in which *H. pylori* is not detected by histochemistry, immunohistochemical testing of *H. pylori* can be used as an ancillary test. In the case of normal histology no immunohistochemical staining should be performed [78].

***MENA region***—Comparative studies of different diagnostic methods have been conducted locally and regionally, but to date, there are no local consensus guidelines.

In a 1996 study conducted in Jeddah, Saudi Arabia, Zaman et al. reported that most culture-positive samples also gave positive results in RUT (94%) [79]. However, they found that serology gave positive results for only 82% and 79% of the culture- and histology-positive samples, respectively. Therefore, serology was unreliable as a single diagnostic method. These findings were in contradiction with another study conducted at King Abdulaziz University Hospital in Jeddah, where Akbar et al. found that serology was more accurate to confirm positive results in histology-positive samples (n = 341) than RUT or culture [80]. However, this study also found that serology, RUT, and culture were less sensitive and specific diagnostic methods than histology. When compared to histology, the relative sensitivity of serology, RUT, and culture was 90%, 81%, and 63%, respectively, while their relative specificity was 47%, 92%, and 93%, respectively. In addition, Saber et al. reported that compared to PCR, the relative specificity and sensitivity of an anti-cagA IgG serologic test were 89.6% and 91.6%, respectively [81]. Serologic tests using serum and salivary were also considered in a study conducted in Abha, Saudi Arabia, by El-Mekki et al. [82]. The sensitivity of the serum and saliva tests was 90.5% and 95%, respectively, while their specificity was 84.5% and 70%, respectively. When they compared histology and culture, Zaman et al. found that 60% of the samples (n = 180) were culture-positive, while 87% were histology-positive [79]. More recently, a study conducted in Abha, Saudi Arabia, by Al Humayed et al. reported that culture and histology gave consistent results (whether positive or negative) in 97.4% of the cases (n = 115) [83]. In general, it appears that culture remains more sensitive and specific than histology, partly because the pathologist’s experience and other factors may affect the interpretation of the histologic samples.

The validity of SAT and UBT was only studied recently. In a study conducted in Abha, Al Humayed et al. compared the sensitivity and specificity of culture, SAT, histology, and RUT (CLOtest). They found that compared to culture, the relative sensitivity of SAT, histology, and RUT was 91.9%, 97.5%, and 79.7%, respectively, while their relative specificity was 98.6%, 97.2%, and 97.2%, respectively [83].

A study conducted in Riyadh, Saudi Arabia, by Al-Fadda et al. also compared different diagnostic methods and reported that compared to histology, the relative sensitivity of RUT and UBT was 88% and 85%, respectively, while their relative specificity was 87% and 70%, respectively. It is important to note that for histology the authors of this study used only one biopsy per patient (the gold standard of the study) [84].

In addition, a study conducted in Iran by Kazemi et al. reported surprisingly low values for the sensitivity (89%) and specificity (73%) of UBT [85]. However, the sensitivity and specificity of RUT (93% and 75%, respectively) and SAT (96% and 83%, respectively) appeared to be comparable to other studies. In this study, a patient was considered infected only if at least two diagnostic methods gave positive results. We believe that the low values for UBT could be attributed to methodological errors. In conclusion, this study recommended using SAT, rather than UBT, for the diagnosis of *H. pylori* infection in untreated patients, while pointing the need for follow-up studies. However, an additional Iranian study (n = 125) by Mansour-Ghanaei et al. found high specificity (100%) and sensitivity (94%) for UBT using radioactive ^14^C-urea [86].

In a study conducted in Saudi Arabia, Mohamed et al. compared three diagnostic methods for *H. pylori*: histology, RUT, and culture [87]. Altogether, *H. pylori* was identified by histology in 145 out of 196 cases (73.98%), while the urease test and culture gave positive results in 126 cases (64.29%) and 102 cases (52.04%), respectively.

There is even less literature on the performances of the different diagnostic methods for *H. pylori* in children. Studies conducted in Egypt by Frenck et al. (n = 108) and Iran by Falsafi et al. (n = 430) recommended using SAT and UBT, because of their high sensitivity (>90%) and specificity (>80%). However, they noted that while remaining high, SAT specificity was lower in children aged <6 years (81%) [88,89]. In comparison, these tests showed higher values in an Israeli study by Hino et al. (n = 92) (sensitivity and specificity of 97.5% and 94.7% for SAT, and 100% and 96.9% for UBT) [90]. Data about accuracy of diagnostic methods for *H. pylori* infection in MENA region are summarized (Table 2).

The available studies conducted in the MENA region and comparing several diagnostic methods did not apply a universal gold standard method for *H. pylori* diagnosis. Moreover, there was no record of a single study considering all the available diagnostic methods and, in most cases, the findings of the different studies were inconsistent. Therefore, further studies are needed to assess affordable, noninvasive diagnostic methods, which are particularly recommended for areas with high prevalence of *H. pylori* infection (like the MENA region). In addition, more attention should be paid to the methodology used in these future studies. For example, it is well-accepted that studies should consider the difference in the approach to diagnosis between children and adults and, therefore, future studies involving children must include noninvasive diagnostic methods. Finally, the existing guidelines from the European Society for Pediatric Gastroenterology, Hepatology, and Nutrition or the North American Society for Pediatric Gastroenterology, Hepatology, and Nutrition are not applicable in areas with high prevalence of *H. pylori* infection, such as the MENA region, which further emphasize the need for new local guidelines [66].

## 4. Conclusions

The evidence presented in this review showed that *H. pylori* infection has a high prevalence in the MENA region, reaching extreme rates in several countries. *H. pylori* infection is usually contracted during the first years of life, and risk factors identified by studies across the region included lower socioeconomic status, level of education, age, contamination of food and water, and smoking.

Finally, the various diagnostic methods for *H. pylori* infection need to be carefully evaluated in well-designed studies, which has not been done in the MENA region. This approach will help in establishing cut-off values specific to each area and improving the diagnostic accuracy. Numerous studies and guidelines emphasize the importance of affordable, non-invasive diagnostic methods in developing countries with a high prevalence of *H. pylori* infection. Currently, the diagnostic methods available for screening in these areas include serology, UBT, and SAT. Moreover, tests based on novel antigens, such as FliD, could be used to improve the diagnostic accuracy. Due to the high prevalence of *H. pylori* infection in the MENA region and the role played by the bacteria in most gastroenterology cases, we highly recommend routine screening for *H. pylori* in all the patients admitted in gastroenterology clinics, which is in agreement with the World Gastroenterology Organization Global Guidelines established in 2011. Therefore, concerted effort from researchers and practitioners worldwide is required to establish affordable and accurate diagnostic methods for this purpose.

## Figures and Tables

**Table 1:** Prevalence of *H. pylori* in Middle East and North Africa (MENA) region and the used diagnostic method.

Region	Sample Size (n)	Positive Cases (%)	Method of Detecting *H. pylori*	Study
**Egypt**	286	207 (72.38%)	Serum IgG (ELISA)	[17]
	89	78 (87.6%)	Serum IgG (ELISA)	[18]
	19	10 (53%)	Serum IgG (ELISA)	
	29	29 (100%)		
	41	39 (95%)		
	169	42 (25%)	Serum IgG (ELISA)	[19]
	169	149 (88%)		
**Iran**	593	488 (82%)	Stool antigen (ELISA)	[20]
	961	384 (40%)	Stool antigen (ELISA)	[21]
	458	294 (64.2%)	Stool antigen (ELISA)	[22]
	525	390 (74.2%)	Serum IgG (ELISA)	[23]
	11596	628 (5.3%)	Serum IgA (ELISA)	[24]
		4500 (38.8%)	Serum IgG (ELISA)	
		442 (7.2%)	Serum IgM (ELISA)	
**Israel**	377	271 (72%)	Serum IgG (ELISA)	[25]
	2093 Jewish	946 (45.2%)	Serum IgG (ELISA)	[26]
	1472 Arabs	619 (42.1%)		
**Lebanon**	414	87(21%)	Stool antigen	[27]
**Libya**	360	275 (76%)	Serum IgG (ELISA)	[28]
**Oman**	133	91 (68.4%)	Serum IgA, IgM & IgG (ELISA)	[29]
**Saudi Arabia**	577	380 (66%)	Serum IgG (ELISA)	[30]
	355	134 (37.7%)	Saliva IgG	[31]
	396	201 (51%)	Serum IgG (ELISA)	[32]
	314	86 (27.4%)	Urea breath test	[33]
	132	68 (51.5%)	Urea breath test	[34]
		70 (53%)	Serum IgG (ELFA)	
	456	129 (28.3%)	Serum IgG (ELISA)	[35]
	N/A	40%	Serum IgG (ELISA)	[36]
**Tunisia**	98	81 (82.7%)	N/A	[37]

IgG: immunoglobulin G; IgA: immunoglobulin A; IgM: immunoglobulin M; ELISA: enzyme-linked immunosorbent assay; ELFA: enzymelinked flourescence assay; N/A: not applicable.

**Table 2:** Diagnostic methods used for *H. pylori* infection in MENA region.

Methods	Sample Size	Sensitivity & Specificity
**Histology**	-	Ranges (53–90%)
	115	97.5% sensitivity
		97.2% Specificity
**Fluorescent in-situ hybridization (FISH)**	27	100%
**Culture**	-	>90% sensitivity
		100% specificity
	341	63% sensitivity
		93% specificity
**Rapid urease test (RUT)**	104	85–95% sensitivity
		95–100% specificity
	341	92% Sensitivity
		81% specificity
	115	79.7% sensitivity
		97.2% specificity
	64	88% Sensitivity
		87% specificity
	94	93% Sensitivity
		75% specificity
**Enzyme immunoassay (EIA)**		Ranges (60–100%)
	341	90% Sensitivity
		47% Specificity
**Urea breath test (UBT)**	-	>95%
	64	85% Sensitivity
		70% specificity
	94	89% Sensitivity
		73% specificity
	125	94% Sensitivity
		100% Specificity
	196	Sensitivity 64.29%
	108	>80% sensitivity
		>90% specificity
	430	
**Stool antigen test (SAT)**	108	>80% sensitivity
		>90% specificity
	430	
	-	(48.9–100%) sensitivity
		(87–94.4%) specificity
	115	91.9% sensitivity
		97.6% specificity
	94	96% sensitivity
		83% specificity
